# Unraveling viral drug targets: a deep learning-based approach for the identification of potential binding sites

**DOI:** 10.1093/bib/bbad459

**Published:** 2023-12-18

**Authors:** Petr Popov, Roman Kalinin, Pavel Buslaev, Igor Kozlovskii, Mark Zaretckii, Dmitry Karlov, Alexander Gabibov, Alexey Stepanov

**Affiliations:** Tetra-d, Rheinweg 9, Schaffhausen, 8200, Switzerland; School of Science, Constructor University Bremen gGmbH, 28759, Bremen, Germany; M.M. Shemyakin and Yu.A. Ovchinnikov Institute of Bioorganic Chemistry of the Russian Academy of Sciences, Moscow, 117997, Russia; Nanoscience Center and Department of Chemistry, University of Jyväskylä, 40014, Jyväskylä, Finland; Tetra-d, Rheinweg 9, Schaffhausen, 8200, Switzerland; School of Science, Constructor University Bremen gGmbH, 28759, Bremen, Germany; Tetra-d, Rheinweg 9, Schaffhausen, 8200, Switzerland; School of Science, Constructor University Bremen gGmbH, 28759, Bremen, Germany; School of Pharmacy, Medical Biology Centre, Queen’s University Belfast, Street, Belfast, BT9 7BL Northern Ireland, U.K; M.M. Shemyakin and Yu.A. Ovchinnikov Institute of Bioorganic Chemistry of the Russian Academy of Sciences, Moscow, 117997, Russia; Department of Chemistry, The Scripps Research Institute, 10550 North Torrey Pines Road MB-10, La Jolla, 92037, CA, USA

**Keywords:** cryptic binding sites learning, SARS-CoV-2, Spike glycoprotein S

## Abstract

The coronavirus disease 2019 (COVID-19) pandemic has spurred a wide range of approaches to control and combat the disease. However, selecting an effective antiviral drug target remains a time-consuming challenge. Computational methods offer a promising solution by efficiently reducing the number of candidates. In this study, we propose a structure- and deep learning-based approach that identifies vulnerable regions in viral proteins corresponding to drug binding sites. Our approach takes into account the protein dynamics, accessibility and mutability of the binding site and the putative mechanism of action of the drug. We applied this technique to validate drug targeting toward severe acute respiratory syndrome coronavirus 2 (SARS-CoV-2) spike glycoprotein S. Our findings reveal a conformation- and oligomer-specific glycan-free binding site proximal to the receptor binding domain. This site comprises topologically important amino acid residues. Molecular dynamics simulations of Spike in complex with candidate drug molecules bound to the potential binding sites indicate an equilibrium shifted toward the inactive conformation compared with drug-free simulations. Small molecules targeting this binding site have the potential to prevent the closed-to-open conformational transition of Spike, thereby allosterically inhibiting its interaction with human angiotensin-converting enzyme 2 receptor. Using a pseudotyped virus-based assay with a SARS-CoV-2 neutralizing antibody, we identified a set of hit compounds that exhibited inhibition at micromolar concentrations.

## INTRODUCTION

The coronavirus disease 2019 (COVID-19) pandemic, which started in December 2019, has caused over a million human deaths worldwide and has become a global challenge in the 21st century. Although the closely related coronaviruses severe acute respiratory syndrome coronavirus (SARS-CoV) and Middle East respiratory syndrome had been known and studied for over a decade, humankind turned out to be helpless against a novel strain, SARS-CoV-2. The World Health Organization reported little or no therapeutic effect for some of the most promising anti-COVID drugs: remdesivir, hydroxychloroquine, lopinavir and interferon [1]. Unprecedented scientific collaborative efforts are being made to develop antiviral therapies, emphasizing the need for fast and efficient response tools to fight viruses at the molecular level. Computational structure-based drug design approaches are matured to high-level precision and take a relatively short time to be applied for a drug target of interest [2]. Unfortunately, target and binding site identification is not straightforward and can arguably be considered one of the most challenging and critical parts of the drug discovery campaign [3, 4]. Generally, the binding site detection methods can be divided into sequence- and structure-based approaches. The sequence-based approaches [5–8] do not take into account protein structure and dynamics, thus, are not applicable to detect binding site opening. The structure-based approaches can be further divided into several categories. The template-based methods screen the query protein against a database and identify regions similar to known binding sites [9–13]. These methods strongly rely on the constructed database of known binding sites and, thus, can detect only similar binding sites in the target protein; moreover, as the database grows, the screening becomes more time-consuming. The geometry-based methods typically utilize information about protein shape [14–19], but miss physicochemical information related to the binding site, unless specifically taken into account. The energy-based methods typically aim to find low-energy regions as potential binding sites using molecular probes [20–23] or analyzing residue dynamics [24]. The classical machine learning approaches utilize sequential and/or structural features to classify amino acids as binding or non-binding [25–29] and strongly rely on the dataset construction and calculated features to describe binding sites. Most recently, due to the rapid accumulation of structural data and the development of deep learning, new deep learning-based methods for binding site identification emerged, which process structural data using graph [30–32] or convolutional [33–38] neural networks. In most cases, these methods show higher accuracy and computational speed, compared with the other types of structure-based approaches, though lack interpretability. Typical obstacles in binding site identification include pitfalls related to protein (i) flexibility, (ii) druggability, (iii) accessibility and (iv) mutability. First, protein flexibility is crucial in drug discovery [39], and a binding site may be present or absent in a given three-dimensional structure; hence, there is a risk of overlooking a relevant binding site or detecting a fleeting and irrelevant binding site [35]. Secondly, not every detected binding site is ‘druggable’, which refers to whether it is possible to make a drug that modulates protein function upon binding that site [4]. Thirdly, the binding site must be accessible to a potential drug; for example, viral proteins can be glycosylated, hence shielding their surface from drug binding [40]. Lastly, viral proteins adapt through amino acid substitutions; therefore, a binding site in one viral strain can be modified or eliminated in another strain [41]. Another essential concern is that for newly discovered binding sites, there is typically a lack of understanding of the modulation mechanism of a potential drug molecule, which limits drug design against novel viral strains or for personalized medicine purposes [42]. Computational approaches that consider the issues mentioned above would help reduce the high rate of false-positive drug target binding sites [43, 44] and facilitate a faster social response in case of future pandemics. Here, we rationalize viral target identification by considering the flexibility, druggability, accessibility and mutability of the protein target, as well as the putative mechanism of action of a potential drug. We used spike glycoprotein S (Spike) as the protein target that covers spherically shaped SARS-CoV-2 virions [45]. Spike has homotrimeric architecture and consists of three subunits responsible for binding to the host cell and merging cellular-viral membranes [46, 47]. One subunit contains the receptor-binding domain (RBD) that undergoes large conformational transitions from the closed (down RBD conformation, PDB: 6VXX [45]) to the open (up RBD conformation, PDB: 6VSB [48]) Spike states. In the open conformation, the virus is capable of binding to the peptidase domain (PD) of angiotensin-converting enzyme 2 (ACE2) with one of its subunit RBDs followed by the fusion process [49, 50]. One of the strategies to prevent viral infection is to design a protein–protein interaction inhibitor that directly blocks the RBD–PD interaction interface [2, 51]. However, such inhibitors could be challenging to design because of highly glycosylated Spike [40, 52]. Another concern is that RBD comprises highly variable amino acid residues, potentially making identified blockers ineffective against different viral strains. Finally, direct RBD–PD inhibitors may affect normal ACE2 function, leading to side effects upon binding to it. Therefore, drugs targeting more accessible and conservative regions in the Spike structure would be safer and have broader applicability than direct RBD–PD inhibitors; many experimental and computational efforts are being made to describe such a distinct region ([41, 53–60], to name a few).

In this study, we identified a vulnerable region in the Spike trimer structure that could be used to allosterically inhibit RBD–PD interactions by preventing the closed-to-open conformational transition of Spike. We analyzed the long-range molecular dynamics (MD) trajectories of Spike using a spatiotemporal deep learning-based approach [35] and selected conformation- and oligomer-specific cryptic binding sites based on the putative mechanism of action and structure-based criteria. Namely, the detected binding site is formed by two Spike subunits: it shares amino acid residues with the RBD of one subunit, and it is present in the closed but not the open conformation of Spike. The binding site is accessible to small molecules and free from glycans. We applied sequence-based and 3D structure-based network analysis to show that the amino acid residues forming the binding site are more conserved and less tolerant of mutations than those in the RBD, indicating a broader application of potential drugs targeting this binding site against viruses from the Coronaviridae family. We further performed virtual ligand screening to select putative binding candidates and compared the flexibility of ligand-free and ligand-bound Spike conformations using MD simulations to identify molecules that stabilize Spike in the closed conformation. Finally, we tested the most promising compounds *in vitro* and confirmed viral inhibition for several compounds in the micromolar concentration range using neutralizing antibody by a pseudotyped SARS-CoV-2 S virus-based assay [61]. Therefore, we hypothesize that ligands bound to the detected binding site could lock Spike in the closed state conformation, preventing the association of the virus with the host cell.

## RESULTS AND DISCUSSION

**Figure 1:**
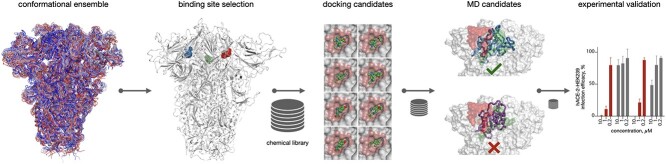
A schematic illustration of the workflow of this study.

This section is organized as follows. First, we described the target binding site selection criteria and its identification. Then we presented the binding site druggability analysis by means of molecular docking of the drug-like compounds into the selected binding site. This is followed by the MD simulations of the top-selected compounds with the hypothesis that an interesting compound would stabilize RBD. Next, we performed virtual ligand screening of a large chemical library, and the most promising hit candidates were validated *in vitro*. Finally, we presented the amino acid residue variability analysis of the identified binding site. Figure 1 schematically demonstrates the proposed approach.

**Figure 2:**
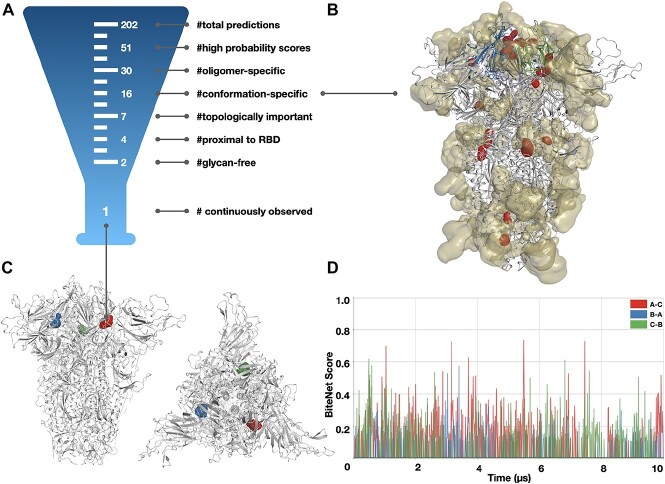
Target binding site identification pipeline. (A) Filtering steps to select the final candidate from the putative binding site predictions (B) Top 16 binding sites predicted in the Spike structure. The predictions are shown with red contours; the glycan densities are shown with yellow transparent surfaces. (C) Side and top view of the closed Spike conformation and the three binding sites corresponding to the three pairs of interacting subunits shown with red, green and blue contours. (D) BiteNet probability scores obtained for the selected binding sites along the 10 $\mu $s MD trajectory.

To allosterically inhibit the RBD–PD interactions, we searched for a vulnerable region in the Spike structure involved in the conformation transition from the closed to the open state. Such a region can be exploited for drug discovery to disrupt the conformational transition, hence inhibiting viral activity. To locate a binding site in the Spike trimer structure that could be used to lock it in the closed state conformation, we searched for a spatial region that (i) involves two or three subunits of the spike (oligomer-specific); (ii) is observed in the closed but not in the open conformation (conformation-specific); (iii) is located near the RBD; (iv) can fit drug-like molecules; (v) is not sheltered by glycan molecules; (vi) is not highly prone to mutations. The first three criteria aim to select a region involved in RBD movement; the fourth and fifth criteria ensure that this region can be exploited for drug discovery; and the last criterion aims to expand the applicability of potential drugs across different viral strains. To detect binding sites satisfying those criteria, we applied BiteNet [35], a deep learning approach for the spatiotemporal identification of druggable binding sites, to the 10 ${\mu }$s MD simulation trajectories by D.E. Shaw Research for the Spike structure in the closed and prefusion states [60] (see Methods). More precisely, we converted the MD trajectories into voxel grids, where each voxel stores atomic densities for different atom types (sulfur, amide nitrogen, aromatic nitrogen, guanidinium nitrogen, ammonium nitrogen, carbonyl oxygen, hydroxyl oxygen, carboxyl oxygen, sp2 carbon, aromatic carbon and sp3 carbon), as the input to the BiteNet 3D convolutional neural network [62]. The network was rigorously trained on atomic structures from the Protein Data Bank (https://www.rcsb.org) to recognize binding sites given the three-dimensional structure of a target, such that the output of the network corresponds to the binding site center along with the probability score for each binding site. Out of 202 putative predictions, 51 passed the probability score thresholds; of these 51, 30 binding sites comprised amino acid residues from the two or three Spike subunits. This was followed by the conformation-specific filter that kept 16 putative binding sites present in the closed but not prefusion conformation (see Figure 2B and Supplementary File 1). The subsequent filter left seven putative binding sites with a median topological importance higher than that calculated for Spike (see Methods). Among these, three are located in the regions occupied by glycans, thus yielding four prefinal candidates. Taking into account the proximity of the RBD domain left us with only two remaining binding sites. Finally, along the 10 ${\mu }$s MD trajectory, one binding site was present in 52.5% of frames (2630 out of 5005), while the other was present in only 5% (250 out of 5005) (see Figure 2A,C). The most promising detected binding site is formed by the two neighboring subunits and comprises amino acid residues from the RBD of one subunit (see Figure 2C). We observed such a binding site for each pair of interacting subunits, and only one binding site, which corresponds to the RBD in the up conformation, was collapsed in the prefusion state. The BiteNet probability score for this binding site varies from 0.0 to 0.7 across the MD trajectory (see Figure 2D); therefore, it can be overlooked in the static structure of Spike. On the other hand, this binding site is continuously observed along the MD trajectory, indicating that it is indeed a binding site, rather than a fleeting prediction. These observations emphasize the role of MD trajectory analysis in binding site identification.

**Figure 3:**
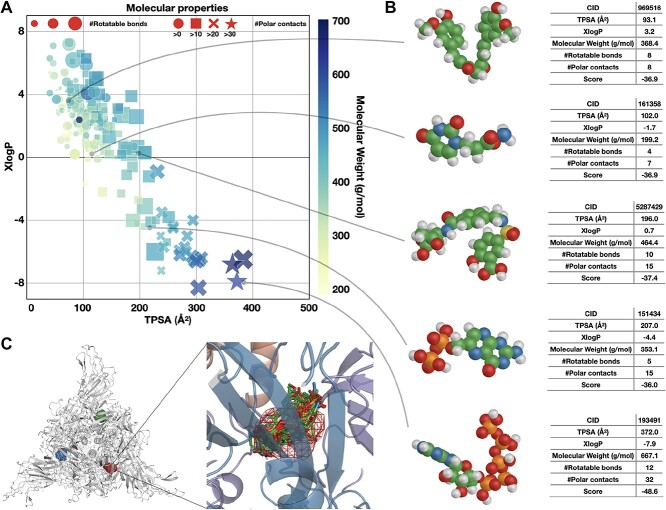
Drug-like molecules identified from molecular docking. (A) Distribution of molecular properties for $209$ top-scored compounds, including topological polar surface area (TPSA), predicted octanol-water partition coefficient (XlogP), molecular weight, number of rotatable bonds and number of potential polar contacts, defined as the sum of the number of hydrogen bond donors and acceptors. (B) Examples of five different compounds; three-dimensional conformers alongside their molecular properties. (C) Superimposed docking poses for the $20$ selected compounds are shown with sticks, and the predicted binding site is shown with mesh.

To analyze the ‘druggability’ of the detected binding site, we used molecular docking to screen ${\sim }$8000 FDA-approved, experimental and investigational compounds retrieved from the DrugBank database [63] using the conformation corresponding to the highest probability score of the binding site (see Methods). We observed ${\sim }$200 drug-like molecules that fit into the binding site with high docking scores (Score ${\leq }{-}30.0$) and formed interactions with both subunits (see Figure 3 and Supplementary File 2). The detected binding site can fit compounds of different molecular weights, octanol-water partition coefficients, topological polar surface areas, numbers of hydrogen bond donors and acceptors and numbers of rotatable bonds (see Figure 3A,B). The high score values for some of the compounds, however, might be artifacts of molecular docking given a large number of possible polar contacts (for example, see Figure 3B, CID: 193491). Therefore, we selected 20 drug-like molecules from the hit list for further investigation, excluding potential artifacts, as well as highly similar compounds. Figure 3C shows superimposed docking poses of these compounds along with the BiteNet predicted binding site. Interestingly, we found experimental structures for seven out of 20 ligands in PDB and observed that root-mean-squared deviation (RMSD) between the experimental and docked binding poses are low ($< 1.9$ Å) for six out of seven cases, suggesting that the obtained binding poses are feasible (see Supplementary File 7).

**Figure 4:**
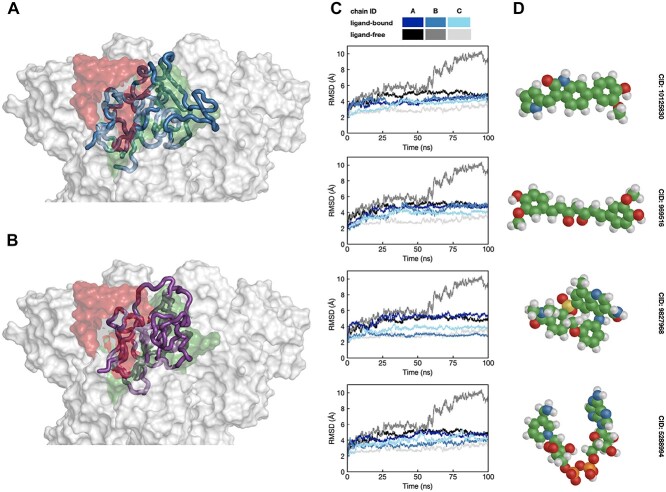
Stabilized RBD in MD simulations of the ligand-bound Spike structure. (A, B) The RBD domain structures corresponding to the last frame of the ligand-bound (CID: 10125830, blue ribbons) and ligand-free (magenta ribbons) simulations. The closed-state (PDB ID: 6VXX) and open-state (PDB ID: 6VYB) structures are shown as green and red surfaces, respectively. (C) RMSD profiles of the RBD domains with respect to the initial structure for the ligand-bound (blue scale) and ligand-free (gray scale) simulations for four of the most stabilizing compounds. (D) Three-dimensional conformers for four of the most stabilizing compounds.

We hypothesize that such small molecules can stabilize bridges, preventing the closed-to-open conformational transition of Spike, thus abolishing viral activity. To support this hypothesis, we ran 100 ns MD simulations of the ligand-bound Spike structures for the 20 selected compounds. Given that the trimer structure is asymmetric and that only one Spike subunit undergoes the closed-to-open conformational transition, we used three compounds placed at three binding sites formed by the A-C, C-B and B-A subunits. Next, we analyzed the RBD flexibility in terms of the RMSD and compared it with the ligand-free MD simulations. We observed that for four out of 20 compounds, the maximal RBD deviation is almost twice as low compared with the ligand-free simulations (see Figure 4A,B). More precisely, the RBD of one of the subunits deviated by 10.0 Å by the end of the ligand-free simulation, while the RMSD values for each RBD in ligand-bound simulations did not exceed 5.0 Å (see Figure 4C and Supplementary File 3). For the other 16 compounds, we observed at least one pair of subunits with similar RMSD values compared with the ligand-free simulation. It is important to note that 100 ns simulations are not enough to observe the RBD transitions from the closed to the fully open state, and longer simulations are required to capture this event [57]. Nonetheless, we did observe the difference between the ligand-bound and ligand-free simulations in terms of the RBD deviation on a smaller scale for four drug-like molecules.

From the results of 1 ms simulation [64], the RMSD values for RBD smaller than 5 Å are observed around 40% of the simulation time, which is consistent with our 100 ns ligand-free simulations. We, thus, hypothesized that ligands for which the corresponding RMSD values are below 5 Å for the entire simulation are more likely to have a stabilization effect and use this hypothesis as one of the criteria to filter hit candidates.

**Figure 5:**
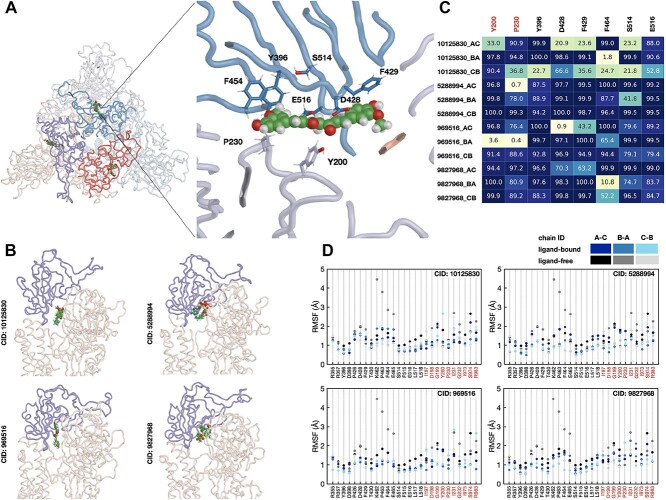
Protein–ligand interactions. (A) Protein–ligand contacts formed within the detected binding site with one of the selected compounds (CID: 969516). Spike is shown with ribbons colored with respect to the chain ID, and the compound is shown with spheres. (B) Interaction interfaces for the top four compounds (one interface per compound) corresponding to the last frames of the MD simulations. (C) The percentage of time the most stable contacts were within 4.0 Åof the ligand during the MD simulations for each interaction interface. (D) RMSF profiles for the amino acid residues observed within 4.0 Å of the ligand during the MD simulations. The amino acid residues of the two subunits are labeled in black and red, respectively.

Analysis of the protein–ligand interactions revealed 27 amino acid residues within 4.0 Å of the ligands across the MD trajectories. To evaluate the flexibility of these 27 amino acid residues, we calculated the root-mean-squared fluctuations (RMSFs) across the MD trajectories, and Figure 5D shows the obtained RMSF profiles for the ligand-bound and ligand-free simulations (see Supplementary File 4 for the RMSF profiles corresponding to all 20 ligands). We observed that the amino acid residues in the ligand-bound trajectories are more stable on average than in the ligand-free MD trajectories, especially the K462-P463-F464-E465 region of the RBD domain, where the RMSF value is at most 2 Å for the ligand-bound MD trajectories. Among these residues, we observed eight that form and maintain close contacts with a ligand during the simulations, namely, Y200 and P230 of one subunit and Y396, D428, F429, F464, S514, and E516 of the other subunit (see Figure 5A). The ligands form hydrophobic contacts with Y200 and P230 of one subunit, and Y200 also forms pi-stacking interactions and hydrogen bonds via its phenolic oxygen. For the other subunit, one can note hydrophobic contacts formed with Y396 and F454, as well as pi- and T-stacking interactions and hydrogen bonds formed by E516 with the ligands. Figure 5C shows the percentage of time these amino acid residues were within 4.0 Å of the ligand for each interaction interface during the MD simulation.

**Figure 6:**
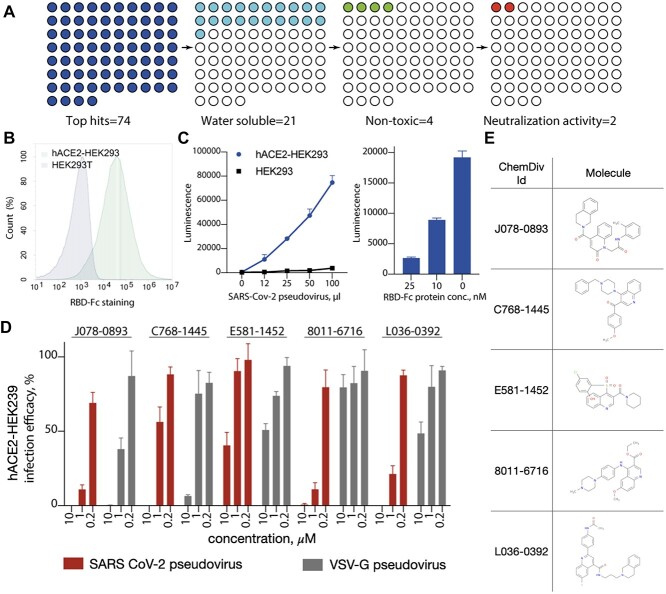
Experimental validation of viral inhibition. (A) Out of 74 compounds selected from virtual ligand screening, 21 had good solubility, four compounds were nontoxic and two demonstrated viral neutralization activity. (B) Binding of pseudotyped virus to hACE2 exposed on hACE2-HEK293 cells. (C) Luciferase signal with respect to the pseudovirus concentration for HEK293 and hACE2-HEK293 cells. (D) Infection efficacy for the top five compounds at 0.2, 1 and 10 micromolar concentrations for SARS Cov-2 pseudovirus as well as VSV-G pseudovirus as a control. (E) Chemical formulas of the tested candidates.

In the next step, we ran structure-based virtual ligand screening of the ChemDiv chemical library (1.5 M compounds, https://www.rcsb.org) using the ICM docking suite (https://www.molsoft.com) and selected the top 74 compounds based on the docking score, estimated physicochemical properties and visual inspection. The top 74 hit candidates were synthesized and reconstituted in DMSO at a 10 micromolar concentration. Only 21 of 74 compounds were soluble in water at 1-10 micromolar concentrations; these water-soluble compounds were tested for cytotoxicity on HEK293 cells (Figure 6A). After overnight incubation, we found that four compounds (C768-1445, E581-1452, 8011-6716 and L036-0392) were not cytotoxic at 1 micromolar, and one compound was nontoxic at 0.2 micromolar (J078-0893). To evaluate the neutralization activity of the identified compounds, we utilized the SARS-CoV-2 S pseudotyped virus based on an HIV-1 lentiviral packaging system with a luciferase reporter [61]. We confirmed that the pseudotyped virus binds to the human angiotensin-converting enzyme 2 (hACE2) receptor exposed on the hACE2-overexpressing HEK-293T (hACE2-HEK293) (Figure 6B) and that the cell viral load correlates with the luciferase signal and hACE2 specificity (Figure 6C). We observed that five compounds inhibited hACE2-HEK293 cell infection at 10 and 1 micromolar concentrations (Figure 6D,E). However, similar to SARS Cov-2, VSV-G pseudotyped virus control transduction was also affected by J078-0893, C768-1445 and E581-1452 compounds. In contrast, 8011-6716 and L036-0392 demonstrated specific infection inhibition at 1 micromolar concentration. We further ran an MD simulation of the 8011-6716 compound in complex with Spike and observed the stabilization effect, similar to the drug-like compounds.

Thus, using a comprehensive *in silico* pipeline that includes target binding site identification and analysis, molecular docking and MD simulation, we identified compounds that inhibit viral infection by the SARS-CoV-2 S pseudotyped virus *in vitro*. However, we want to stress that although we hypothesize that the identified compounds demonstrate inhibition by means of binding to the detected binding site, there is a possibility that the identified hit candidates infer its inhibition effect by binding to a different binding site or by a different molecular mechanism in general. Therefore, further investigations, such as crystallographic or cryogenic electron microscopy structure determination of Spike in complex with the identified hit candidates, are required to rigorously validate our hypothesis.

**Figure 7:**
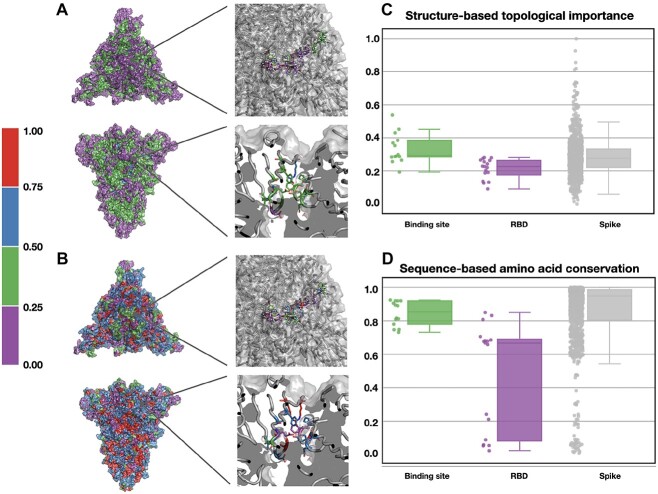
Structure-based topological importance and sequence-based conservation of the binding site. (A, B) Top and side views of the Spike structure colored with respect to the structure-based topological importance and the conservation score of the Spike amino acid residues, respectively. (C, D) Box plots of the structure-based topological importance and Valdar’s conservation score calculated for the binding site, RBD, and exposed amino acid residues of Spike, respectively.

Variability of the amino acid sequence is one of the greatest challenges in drug repurposing and drug discovery for use against viruses. As a consequence, drugs targeting less variable binding regions might be effective across different viral stamps. It was shown that the topological importance of the amino acid residues is critical in accessing mutational tolerance for viral proteins, especially to predict vulnerable epitopes [65]. Accordingly, we applied structure-based network analysis [66] to estimate the topological importance of the Spike trimer (see Methods and Supplementary File 6). We compared regions corresponding to the RBD, which is a common drug target, and the detected binding site (see Figure 7A,B,C). We observed that the detected binding site is endowed with lower mutation tolerance than the RBD (0.35 versus 0.22 for the median topological importance). Moreover, we analyzed the amino acid sequence conservation profiles of the Spike proteins from the coronavirus family (see Methods and Supplementary File 5 for the constructed multiple sequence alignments). We calculated the Valdar’s conservation scores [67] and observed that the detected binding site is more conserved among the coronavirus family than the RBD domain or the set of exposed amino acid residues of Spike (see Figure 7D,E,F and Supplementary File 6). Altogether, these results demonstrate that the detected binding site corresponds to the vulnerable region in the Spike structure of the coronavirus family and indicates a larger applicability domain for drugs targeting it than those targeting the RBD.

To summarize, we presented a computational pipeline for rational drug target binding site identification in viral proteins. We applied the pipeline to the SARS-CoV-2 spike glycoprotein S and identified the vulnerable region, which is more conserved and topologically important than the RBD. Molecular docking and MD simulations revealed drug-like compounds that stabilize the RBD in the closed state, indicating the possibility of inhibiting RBD–PD interactions allosterically, hence abolishing viral activity. The subsequent *in silico* ligand screening and *in vitro* testing of the most promising compounds helped to identify compounds that demonstrate viral inhibition effects at micromolar concentrations.

## METHODS

### Binding site identification

To predict a vulnerable region in Spike, we considered 10 ${\mu }$s MD trajectories of the closed- and pre-fusion Spike states made by D.E.Shaw Research [60]. We used the GROMACS trjconv utility to split trajectory into a set of.pdb files [68] that contain only protein chains. Then we applied BiteNet [35] to the closed-state MD simulation using the probability score threshold of 0.01 resulting in 202 putative binding site candidates. This was followed by filtering predictions using the clustering score threshold of 0.01, leading to 51 predictions. In the next step, we filtered out only binding sites that are within 8 Å from only one protein chain yielding 30 candidates. This was followed by filtering predictions also observed in the prefusion-state MD simulation; thus, we obtained top 16 binding sites. Then, we calculated the topological importance for each residue, and took seven binding sites with the median topological importance higher as compared with the entire Spike. Next, we considered binding sites within 8 Å from the RBD region starting from 350th amino acid residue, resulting in four binding sites. Two predictions were sheltered by glycans and were filtered out; the densities of glycans were calculated for all conformations of the closed-state Spike MD trajectory using VMD [69]. Finally, we compared the time ratio, a binding site was observed in the MD simulation, and selected the one corresponding to a higher fraction as the final candidate (52% versus 5%).

### Amino acid conservation analysis

We retrieved amino acid sequences corresponding to the Spike protein from the GISAID [70] database (version March 2023) as well as the reference sequence from the UniProt [71] (UniProt ID: P0DTC2). We kept only unique protein sequences to avoid bias toward overrepresented strains and disregarded sequences with lengths out of the Q1-Q3 quartile range (Q1=1270, Q3=1273). Finally, we substituted all non-standard or unknown amino acid residues with ‘X’ and kept only sequences that contain less than 60 ‘X’, resulting in 1 144 383 sequences. We aligned all the protein sequences to the reference sequence using mafft [72]. Finally, we calculated the Valdar’s conservation score[67] for each amino acid residue position.

### Topological importance analysis

The topological importance was calculated using the structure-based residue interaction network approach [65]. We used the starting frame of the closed-state MD trajectory for the analysis. We disregarded glycans and water molecules beyond 3 Å from the protein chains. We skipped the first steps of the network workflow aimed to add hydrogen atoms [66]. The atom names for hydrogen and water molecule oxygen were renamed according to the network workflow.

### Molecular docking

We retrieved investigational, experimental and approved drug molecules from the DrugBank database [63] library in the SMILES format and applied the standardization procedure according to the ChEMBL structure standardization pipeline [73] yielding $8282$ compounds. We want to note that we refer to the PubChem compounds as to the standardized versions of these compounds; however, one should keep in mind that the standardization may change a molecule, particularly stereoisomerization. Then we generated three-dimensional conformations and assign partial charges for each compound using the semi-empirical PM3 optimization method implemented in the ORCA software package [74]. This procedure yielded 8096 3D conformers, which we converted to the MOL2 format for molecular docking. For the hit identification, we used the ChemDiv chemical library of ${\sim }$1.5M compounds retrieved from https://www.rcsb.org. As the protein structure, we used the Spike conformation from the 10 ${\mu }$s trajectory corresponding to the highest probability score of the binding site of interest. We pre-processed the structure by optimizing side-chain rotamers using Monte Carlo optimization and the MMFF-94 force field. A rectangular box enclosing amino acid residues within 8 Å of the binding site center with an additional 8 Å margin was used as the sampling space for molecular docking. The protein structure was presented as smoothened grid potentials, while the docking simulations sampled ligand conformations in the internal coordinate space using biased probability Monte Carlo optimization [75] implemented in ICM-Pro by MolSoft www.molsoft.com with the sampling parameter (docking effort) set to 30. Finally, we constructed the docking hit list ranked with respect to the docking score of the drug-like candidates and selected the top 20 compounds, excluding highly similar and those with multiple PO4 groups, for further analysis.

### MD simulations and analysis

MD simulations were performed using the GROMACS 2018.6 software [68]. The starting structure was prepared by merging the CHARMM-GUI glycosylated structure [76] with structures by D. E. Shaw Research [60]. The parameters were taken from CHARMM-GUI [76] and reduced to atoms present in structures provided by D. E. Shaw Research [60]. The starting coordinates of the spike protein were pulled to the coordinates of the Spike conformation used for molecular docking by applying position restraints on spike backbone atoms with force constant 10 000 $kJ/mol/nm^2$ for 1 ns. This resulted in a glycosylated structure with backbone atoms RMSD of $< 1$Å from the structure used for molecular docking. The MD parameters for 21 of the selected compounds were obtained from Swissparam [77]. The compounds were placed into the binding sites at all three interaction interfaces. For that, the compound positions from the molecular docking were aligned with respect to the other interfaces using MDAnalysis [78]. Overall, 22 systems were prepared, including one ligand-free and 21 ligand-bound. For all the systems, periodic boundary conditions were applied. A constant temperature of 303 K and pressure of 1 bar were maintained using Nose–Hoover thermostat [79] with time constant of 1.0 ps and isotropic Parrinello–Rahman barostat [80] with a time constant of 5.0 ps. The Leanard–Jones cutoff was set to 1.2 nm, and Lennard–Jones interactions were smoothly switched to zero at distances higher than 1.0 nm. Electrostatic interactions were treated with the particle mesh Ewald method [81]. A leapfrog integrator was used with an integration step of 2 fs. The bond distances and bond angles of water molecules were constrained using the SETTLE algorithm [82], and all other bond distances were constrained using the LINCS algorithm [83]. Prior to all simulation runs, the potential energy was first minimized using the steepest descent method, followed by 125 ps equilibration MD runs. The production runs for 100 ns were performed, and frames were saved every 100 ps.

### Cell lines

The 293T cells were cultured in DMEM (Gibco, Catalog #10-566-016) supplemented with 10% FBS (HyClone, Catalog #SH30079.03), 10 mM HEPES (Gibco, Catalog #15-630-130), 100 U/ml penicillin, 100 microgram/ml streptomycin and 2 mM GlutaMAX (Gibco, Catalog #35-050-079).

### Cloning

The plasmid vectors pCG1-SARS-2-S with a gene of codon-optimized S-protein with $\Delta 19$ and furin cleavage site mutations (R682G or R685K) [84] and pCG1-hACE2 were kindly provided by Prof. Dr. Dmytriy Mazurov (Institute of Gene Biology RAS). The DNA fragment coding for the hACE2 were synthesized and cloned into the dual promoter vector pCDH511b (CMV MCS/EF1a eGFP) under the control of the CMV promoter. The soluble protein expression vectors were generated by cloning of the SARS-2-S and hACE2 extracellular domains into the pFUSE-Fc vector (Invivogen).

### SARS Cov-2 and VSV-G pseudotyped viruses

The lentiviral particles were prepared by co-transfection of HEK293T cells with lentiviral vector coding for firefly luciferase (pCDH-CMV-LUC-EF1 Hygro, Addgene #129437), GAG and Rev packaging plasmids combined with VSV-G or pCG-SARS-2-S envelop plasmids. Supernatants containing the virus were collected at 72h post-transfection and lentiviruses were concentrated with the Lenti-X Concentrator (Takara, Catalog #631232). Aliquots of viruses were snap-frozen in liquid nitrogen and stored at -80C. Pseudotyped virus aliquots were titrated on hACE2-HEK293T cells and assessed by luciferase assay.

### Compounds neutralization activity against pseudovirus

hACE2-HEK293 cells were generated by transduction of HEK 293 cells with lentiviruses coding for hACE2 and GFP (pCDH511b CMV hACE2/EF1a eGFP). Then, hACE2-HEK293 cells, which express ACE2 receptor, were infected with pseudovirus expressing the VSV-G or SARS Cov-2 and luciferase reporter gene in the presence and absence of serial dilutions of testing compounds. Viral entry to the cells was quantified using the Bright-Glo™ Luciferase Assay System (# E2620, Promega). The dose–response curves were plotted with the relative luminescence unit against the sample concentration.

### RBD and hACE2 proteins expression and purification

The SARS-2-S-Fc and hACE2-Fc were expressed using the FreeStyle 293 Expression System. Four days after transfection media was collected, centrifuged 15 min 350 g at RT, and filtered through a 0.22$\mu{m}$ filter. The supernatants of cell culture containing the SARS-2-S-Fc and hACE2-Fc were concentrated and Fc-fusion proteins were captured by HiTrap™ Protein G HP (Amersham). Then, proteins were concentrated and buffer-exchanged to PBS by using Centricon Ultra 30K (Merck).

### Flow cytometry

Cells were washed with PBS, resuspended in 100 microL of PBS with 0.5% BSA at a concentration of 106 cells per mL, labeled with ACE2-Fc or SARS-2-S-Fc at a final concentration of 1 microgram/mL, washed with PBS and was secondary staining with anti-IgG4 Goat anti-Human, DyLight™ 650 (SA510137, Invitrogen) according to the manufacturer’s recommendations, washed, and analyzed using NovoCyte 2060 flow cytometer (ACEA Biosciences, USA). Data were analyzed with NovoExpress Software (ACEA Biosciences).

### Visualization

We used PyMol v.2.3.5 [85] to produce structural images, and matplotlib v.3.3.0 [86] and plotly v.4.12.0 [87] python libraries, and the Mathematica v.10.1 package [88] to produce data plots.

## CONCLUSION

In this study, we introduced a computational approach to identify vulnerable regions in viral proteins, specifically focusing on SARS-CoV-2 spike glycoprotein S. By considering protein dynamics, accessibility and mutability and the putative mechanism of action of drugs, we aimed to detect promising binding sites for potential therapeutic interventions. Our analysis of MD trajectories revealed a conformation- and oligomer-specific glycan-free binding site comprising topologically important amino acid residues proximal to the RBD. Through virtual ligand screening, we identified several promising hit candidates; to validate their potential, we conducted *in vitro* assays, confirming their efficacy in inhibiting the virus. We postulate that these ligands, when bound to the identified binding site, have the ability to lock the Spike protein in the closed conformation, thereby impeding viral association with host cells. Overall, our study demonstrates the effectiveness of our structure- and deep learning-based approach in identifying drug binding sites and presents potential drug candidates for inhibiting the interaction between SARS-CoV-2 spike glycoprotein S and the hACE2 receptor. The presented computational approach could help to prepare better for the next pandemic by identifying the most relevant viral drug target binding sites for drug discovery and design.

**Key Points**

- Deep learning-based workflow enables binding site detection in viral drug targets.
- SARS-CoV-2 Spike hides potential oligomer- and conformation-specific binding site.
- This binding site comprises highly conservative amino acid residues, thus, it is a vulnerable region of the coronavirus family.
- Ligands targeting the identified binding site could stabilize the closed conformation of Spike, thus, inhibiting its activity.

## Supplementary Material

Supplementary_Data_bbad459

## Data Availability

The data underlying this article are available in the article and in its supplementary materials available athttps://zenodo.org/records/8400118.

## Author Biographies

**Petr Popov** is an Adjunct Professor at Constructor University of Bremen. His research interests are in numerical optimization methods for drug discovery.

**Roman Kalinin** is a Ph.D. student at M.M. Shemyakin and Yu.A. Ovchinnikov Institute of Bioorganic Chemistry of the Russian Academy of Sciences. His research interests are in CART-cell technologies for targeted cancer therapy.

**Pavel Buslaev** is a postdoctoral researcher at the NanoScience Center of the University of Jyväskylä. His research interests are in molecular dynamics simulations.

**Igor Kozlovskii** is a PhD student at Constructor University of Bremen. His research interests are in deep learning for drug discovery.

**Mark Zaretckii** is a PhD student at Constructor University of Bremen. His research interests are in deep learning for drug discovery.

**Dmitry Karlov** is a postdoctoral researcher at the Queen's University Belfast, U.K. His research interests are in computational chemistry and drug discovery.

**Alexander Gabibov** is the Director of M.M. Shemyakin and Yu.A. Ovchinnikov Institute of Bioorganic Chemistry of the Russian Academy of Sciences. His research interests are biocatalysis, immunochemistry, and neurobiology.

**Alexey Stepanov** is Staff Scientist at Scripps Research Institute. His research interests are in the design of new types of anticancer therapies based on chimeric antigen receptor therapy.
